# In Vitro Analysis of the Effects of ITER-Like Tungsten Nanoparticles: Cytotoxicity and Epigenotoxicity in BEAS-2B Cells

**DOI:** 10.3390/nano9091233

**Published:** 2019-08-30

**Authors:** Chiara Uboldi, Marcos Sanles Sobrido, Elodie Bernard, Virginie Tassistro, Nathalie Herlin-Boime, Dominique Vrel, Sébastien Garcia-Argote, Stéphane Roche, Fréderique Magdinier, Gheorghe Dinescu, Véronique Malard, Laurence Lebaron-Jacobs, Jerome Rose, Bernard Rousseau, Philippe Delaporte, Christian Grisolia, Thierry Orsière

**Affiliations:** 1 CNRS, IRD, IMBE, Avignon Université, Aix Marseille Université, 13005 Marseille, France; 2 CNRS, IRD, INRA, Coll France, CEREGE, Aix Marseille Université, 13545 Aix-en-Provence, France; 3 CNRS, LP3, Aix Marseille Université, 13005 Marseille, France; 4 CEA, CNRS, BIAM, Aix Marseille Université, 13108 Saint Paul-Lez-Durance, France; 5 CEA, IRAMIS UMR NIMBE, Université Paris Saclay, 91191 Gif-sur-Yvette, France; 6 LSPM, Université Paris 13, UPR 3407 CNRS, 93430 Villetaneuse, France; 7 CEA, SCBM, Université Paris Saclay, 91191 Gif-sur-Yvette, France; 8 INSERM, MMG, Aix Marseille Université, 13005 Marseille, France; 9 INFLPR, 409 Atomistilor Street, Magurele, 77125 Bucharest, Romania; 10 CEA, IRFM, 13108 Saint Paul lez Durance, France

**Keywords:** tungsten, nanoparticles, tritiated particles, in vitro testing, cytotoxicity, micronuclei formation, DNA damage, epigenetics, DNA methylation, BEAS-2B cells.

## Abstract

Tungsten was chosen as a wall component to interact with the plasma generated by the International Thermonuclear Experimental fusion Reactor (ITER). Nevertheless, during plasma operation tritiated tungsten nanoparticles (W-NPs) will be formed and potentially released into the environment following a Loss-Of-Vacuum-Accident, causing occupational or accidental exposure. We therefore investigated, in the bronchial human-derived BEAS-2B cell line, the cytotoxic and epigenotoxic effects of two types of ITER-like W-NPs (plasma sputtering or laser ablation), in their pristine, hydrogenated, and tritiated forms. Long exposures (24 h) induced significant cytotoxicity, especially for the hydrogenated ones. Plasma W-NPs impaired cytostasis more severely than the laser ones and both types and forms of W-NPs induced significant micronuclei formation, as shown by cytokinesis-block micronucleus assay. Single DNA strand breaks, potentially triggered by oxidative stress, occurred upon exposure to W-NPs and independently of their form, as observed by alkaline comet assay. After 24 h it was shown that more than 50% of W was dissolved via oxidative dissolution. Overall, our results indicate that W-NPs can affect the in vitro viability of BEAS-2B cells and induce epigenotoxic alterations. We could not observe significant differences between plasma and laser W-NPs so their toxicity might not be triggered by the synthesis method.

## 1. Introduction

Thermonuclear fusion could potentially represent an unlimited carbon-free source of energy. The International Thermonuclear Experimental fusion Reactor (ITER) project is—at present—exploiting this hypothesis. Based on the current configuration of the ITER fusion plant, tungsten (W) will be the main component of the divertor of the tokamak reactor, the place where the maximum of energy is deposited. Among other materials, W has been chosen thanks to its robustness, its elevated melting point and its resistance to erosion by the tokamak plasma [1]. Nevertheless, tritiated particles will be formed with a theoretical size ranging from tens of nm to tens of micrometers. The safety of reactor’s workers and of those living in the nearby area is ensured by High Efficiency Particulate Air (HEPA) filters. Their function is to prevent environmental contamination and occupational or accidental exposure to W particles. However, HEPA filters have a lower retention capability for particles in the 100–500 nm range [2], so a fraction of the ITER-derived W nanoparticles (W-NPs), might escape the reactor in the case of Loss-Of-Vacuum-Accident (LOVA) and disperse into the environment.

Even if W is used in different applications ranging from military use to diagnostics and therapeutic medical devices, from electronics to tanning processes, there is no consensus on its toxicity mechanisms mainly due to its possible redox transformation. Up to now, W has been studied under different forms such as tungsten carbide (WC) alloys doped with cobalt (WC-Co), sodium tungstate (Na_2_WO_4_), tungsten (VI) oxide nanoparticles (WO_3_NPs), and metallic W.

The soluble Na_2_WO_4_ (W^6+^) was able to induce apoptosis and cell cycle blockage in human peripheral lymphocytes [3] and, by enhancing the expression of genes related to cancer and inflammation, to alter gene expression in the bronchial human-derived BEAS-2B cell line [4]. WO_3_NPs (W^6+^) impaired the viability of human-derived lung alveolar A549 cells, enhanced DNA damage and micronuclei formation, as well as ROS production and apoptosis [5].

In contrast, more information is available on WC-Co alloys, classified as probably carcinogenic in humans (Group 2A) by the International Agency for Research on Cancer [6]. In BEAS-2B cells, WC-Co was shown to reduce the cellular viability in a time and concentration related manner. When comparing micrometric and nanometric particles, it was observed that only nano-WC-Co caused actin rearrangements and was internalized by BEAS-2B cells [7]. The very same WC-Co particles were reported to impair the cellular viability and increase the secretion of pro-inflammatory cytokines more severely in BEAS-2B than in co-cultures with a monocytic cell line as THP-1 cells or in THP-1 monocultures. These results were explained by suggesting that THP-1 cells might somehow protect the epithelial BEAS-2B cells from the toxic mechanisms exerted by WC-Co [7]. A cell-related effect of WC-Co was also observed in other studies [8,9,10]. Bastian et al. reported that the A549 cell line was less affected by WC-Co particles than other cell types such as the intestinal Caco-2 and the skin HaCaT cells, and that the presence of cobalt ions enhanced the toxicity of WC particles [8]. Moche et al. showed that WC-Co is a suitable candidate as positive control in genotoxicity assays, but that cell type and exposure length play a key role. In fact, while WC-Co particles did not provide satisfactory positive results in L5178Y mouse lymphoma cells, freshly isolated human lymphocytes were more sensitive, especially at short exposures (4 h) when primary DNA damage was studied, and at long exposures (24 h) when chromosome breakage or loss was considered [9]. Finally, Paget et al. reported that A549 cells underwent less severe cytotoxicity, DNA damage, and cell cycle arrest compared to Caki-1 kidney cell line and Hep3B liver cells, although similar WC-Co internalization was measured [10]. These three above mentioned publications [8,9,10] suggest that the mechanisms triggering WC-Co cyto- and geno-toxicity are the production of reactive oxidative species (ROS) and the different antioxidant capability in each of the cell types investigated. As previously demonstrated, in fact, Co provides the electrons that deposit on WC particles, thus reducing the oxygen on their surface and generating ROS [11].

The less studied form of W is the metallic one and, to our knowledge, only one publication is available. Machado and coauthors investigated, on A549 lung cells, the effects of mixtures of ballistic debris containing W (W-Ni-Co and W-Ni-Fe) as well as of nanosized and of micrometric metallic W particles [12]. Their results showed that the metallic W nanoparticles exerted severe cytotoxicity but not as significantly as the mixture of ballistic debris, and that the micrometric metallic W did not impair cellular viability [12].

Altogether, the literature suggests that W can impair the integrity and the normal functioning of in vitro cellular systems representative of different organ compartments. Nevertheless, the genotoxic and epigenetic effects of W-NPs have not yet been fully investigated.

In this study, we used the bronchial human-derived epithelial BEAS-2B cell line [13,14] to describe the epigenotoxicity of bench synthesized plasma sputtering and laser ablation ITER-like W-NPs. In order to mimic the particles generated by the ITER fusion reactor, W-NPs produced by plasma sputtering and laser ablation were hydrogenated and tritiated. Moreover, the size of our ITER-like W-NPs resulted in the HEPA filters escape range, further enhancing the importance of our study in an occupational risk perspective. Cell viability, evaluated via the quantification of the adenosine triphosphate (ATP) by luminescence, and cytostasis, evaluated via the cytome version of the Cytokinesis Block MicroNucleus assay (CBMN-cyt), showed that both plasma and laser ITER-like W-NPs are toxic to BEAS-2B cells and that the effect was enhanced upon exposure to hydrogenated/tritiated particles. Laser ablation-derived ITER-like W-NPs seem to exert a slightly enhanced micronuclei formation and primary DNA damage, as shown by CBMN-cyt and alkaline comet assay, respectively. Pancentromeric staining revealed that plasma and laser ITER-like W-NPs induced both clastogenic and aneugenic effects. Epigenetics, in contrast, showed that laser and plasma ITER-like W-NPs had no effects on the DNA methylation of BEAS-2B cells. Finally, oxidative stress, which has been proposed as the factor triggering the cytogenotoxicity of other types of W compounds, has been investigated and a significant alteration of the oxidized/reduced glutathione content was detected. Since more than 50% of W exhibits oxidative dissolution, the role of W^6+^ species must be considered in the biological effects.

## 2. Materials and Methods

### 2.1. Reagents

LHC-9 and LHC basal medium, Bovine Serum Albumin (BSA), fibronectin, collagen, PBS, and Trypsin-EDTA were purchased from Thermo Fisher Scientific (Illkirch, France). CellTiter-Glo^®^ Luminescent Cell Viability Assay and GSH-Glo™ Glutathione Assay were purchased from Promega (Charbonnières-les-Bains, France). All other reagents were purchased from Sigma-Aldrich (St. Quentin Fallavier, France).

### 2.2. Particles Synthesis and Suspensions Preparation

Two synthesis methods were used to produce ITER-like W-NPs with different physico-chemical properties: magnetron plasma sputtering and gas condensation (plasma W-NPs), and laser ablation (laser W-NPs). Detailed description of the production set-up and morphology, composition, and crystalline structure were already described [15,16,17]. Given the characteristics of the tokamak that will operate at ITER, particles of irregular shape and of smaller size than those produced by other already existing W-based tokamaks are expected. Plasma sputtering and laser ablation were thus believed to be the two synthesis methods that can produce W-NPs that most likely will resemble those generated during ITER operation.

Plasma-derived ITER-like W-NPs were produced in a cluster source by condensation of the metal vapors supplied into argon gas flow by radio-frequency (13.56 MHz) magnetron sputtering discharge [18]. The metallic clusters produced were collected on a dedicated substrate.

Laser-derived ITER-like W-NPs were produced using laser power density deposition on a tungsten target, a dust production technique with relative simplicity and flexibility was developed in preliminary projects [19] and upgraded for the specific requirements of this study. Absorption of the laser energy in the W metallic structure triggers the excitation of free electrons, and this energy is relaxed in the matrix through a thermic wave conveyed by phonons. The brief and intense heat source at the surface of an ITER grade W sample submitted to the laser impulses allows the formation of particles by two processes: ejection of melted material or accretion of particles in the plasma formed in the heated volume. We used a picosecond laser of wavelength 1064 nm, frequency 10 Hz, pulse energy density 5 J/cm^2^, and pulse duration 50 ps to produce laser W-NPs [15].

The physical and chemical stability of the W-NPs in biological media were assessed by mean of Dynamic Light Scattering (DLS) using the Mastersizer S (Malvern Panalytical; Orsay, France) and dissolution experiments [17]. The chemical analysis of W in the suspension was performed within inductively coupled plasma mass spectrometry (ICP-MS) (Perkin Elmer Nexion 300; Villebon-sur-Yvette, France). H_2_O_2_ solutions at room temperature for 24 h were used to digest the solid forms of W solid forms via a total oxidation. The dissolved W species were isolated by combining two filtration stages at 25 and 20 nm [17].

In the facility where radioactive species were manipulated, all the equipment necessary to investigate the physico-chemical properties and the cyto-epi-genotoxic potential of tritiated W-NPs were not available. We therefore included in our study both the pristine and the hydrogenated forms of laser and plasma W-NPs. Pristine is the form in which W-NPs were bench produced; hydrogenated W-NPs were investigated because they are equivalent to the tritiated ones, but not radioactive. Their lack of radioactivity allowed us to thoroughly study them even in the absence of a dedicated facility.

### 2.3. Cellular Model

The transformed human bronchial epithelial BEAS-2B cell line (ATCC CRL #9609; LGC Standards Sarl, Molsheim, France) was cultured in sterile tissue culture treated flasks or plates pre-coated with LHC basal medium supplemented with BSA (0.01 mg/mL), human fibronectin (0.01 mg/mL), and collagen (0.03 mg/mL). The cultured cells were maintained in LHC-9 medium under standard cell culture conditions (37 °C in 5% CO_2_ at 95% humidity) and passaged before confluence by trypsin (0.25%)-EDTA (2.6 mM).

For the experiments with tungsten, BEAS-2B cells were exposed for 2 h and/or 24 h to increasing concentrations (0–150 µg/mL) of plasma and laser ITER-like W-NPs.

### 2.4. Cytotoxicity Assessment

*Intracellular ATP*. The viability of BEAS-2B cells was indirectly evaluated via the *in vitro* quantification of intracellular ATP produced by metabolically active cells upon exposure to W-NPs (1–150 µg/mL). The CellTiter-Glo^®^ Luminescence Cell Viability Assay was performed as previously described [20]. Data were acquired using a GloMax^®^ Explorer Multimode Microplate Reader (Promega; Charbonnières-les-Bains, France). For each experimental point, three independent assays were performed, each of them in triplicate (*n* = 9). The percentage of cellular viability was compared to the unexposed control cells.

*Cytostasis and cellular replication*. By performing the cytome version of the Cytokinesis Block MicroNucleus (CBMN-cyt) assay [21], adapted to the use with nanoparticles [22], cytostasis and cellular replication were also evaluated. To assess cytostasis, the Cytokinesis Block Proliferation Index (CBPI) was calculated by scoring mononucleated, binucleated, and multinucleated cells in the first 500 living cells analyzed in each sample. CBPI, which indicates the average number of cell divisions completed by the cells, was calculated as follows:

[(1 × number binucleated) + (2 × number multinucleated) + (3 × number multinucleated)]/(500 viable cells).(1)

The percentage of cytostasis was calculated as recommended by OECD in the test guideline 487 [23]:

{100 − 100 × [(CBPI exposed cells-1)/(CBPI control cells-1)]}.(2)

The replication index, which represents the proportion of cell division cycles completed in a treated culture during the exposure period and recovery, was calculated as described in the OECD test guideline 487 [23]:

[(number of binucleated cells) + (2 × number multinucleated cells)]/total exposed cells/ ([(number binucleated cells) + (2 × number multinucleated cells)]/total control cells) × 100.(3)

### 2.5. Genotoxicity Studies: Micronucleus and Alkaline Comet Assay

*Cytokinesis-Block MicroNucleus assay*. To identify chromosome breakage or chromosome loss following exposure W-NPs, the Cytokinesis-Block MicroNucleus assay (CBMN) was performed as described before [20].

Briefly, BEAS-2B cells were seeded onto a two-well Lab-Tek^TM^ II Chamber SlideTM System (Nalgene Nunc International, Villebon sur Yvette, France) and treated with increasing concentrations (0–20 µg/mL) of plasma and laser ITER-like W-NPs. After 24 h exposure, cells were washed and 3 μg/mL cytochalasin B (Sigma Aldrich Chimie Sarl; St. Quentin Fallavier, France) was added to the cultures to block cytokinesis; BEAS-2B cells were kept in culture for an additional 28 h and then cells were fixed with 4% (*v*/*v*) paraformaldehyde in PBS. Mitomycin C (MMC, 0.1 µg/mL) served as a positive control, whereas LHC-9 culture medium as the negative one. Upon permeabilization, the cytoskeleton was stained with phalloidin-tetramethylrhodamine B isothiocyanate (Sigma Aldrich Chimie Sarl; St. Quentin Fallavier, France), while nuclei with DAPI ProLong^®^ Gold antifade reagent (Fisher Scientific; Illkirch, France).

CBMN was performed in duplicate, and slides were blindly scored using an Axio Imager. A2 fluorescence microscope (Carl Zeiss S.A.S; Marly Le Roi, France) at 400× magnification. Micronuclei (MN) were only assessed in binucleated (BN) cells that had completed one nuclear division following exposure to the test compounds [20]. For each experimental condition, the number of binucleated micronucleated (BNMN) cells was scored in 1000 BN cells.

*Comet assay*. To detect the primary DNA damage induced by plasma and laser ITER-like W-NPs in BEAS-2B cells the alkaline comet assay was performed as previously described [20]. BEAS-2B cells, seeded onto pre-coated 12-well plates (BD Falcon; Le Pont de Claix, France), were exposed to W-NPs (1–20 µg/mL) for 2 and 24 h. At the end of the treatment period, cells were washed, trypsinized, resuspended in low melting point agarose, and then spotted onto glass slides pre-coated with 1.6% and 0.8% normal melting point agarose. Cells were then lysed, and the DNA denatured in a MilliQ water solution containing NaOH and EDTA. After electrophoresis (25 V and 300–315 mA), samples were neutralized and dehydrated. Air-dried slides were stained with propidium iodide (PI) right before imaging and data acquisition. Cells incubated only with LHC-9 medium served as negative control, while hydrogen peroxide (110 µM) was used as positive control. Slides, which were prepared in duplicate for each experimental condition, were analyzed under a fluorescence Axio Imager. A2 microscope (Carl Zeiss S.A.S; Marly Le Roi, France) at 400× magnification using the Komet 6.0 software (Andor Bioimaging, Nottingham, UK). DNA damage was expressed as mean % tail DNA ± standard error of the mean (SEM).

### 2.6. Oxidative Stress Evaluation

The induction of oxidative stress in BEAS-2B cells upon exposure to W-NPs (1–20 µg/mL) was detected by performing the GSH/GSSG-Glo™ Assay (Promega; Charbonnières-les-Bains, France), a luminescence-based system suitable to determine the ratio between the reduced glutathione GSH and its oxidized form GSSG in cultured cells. According to the manufacturer instructions, cells were cultured in a 96-well plate and then exposed for 30 min to plasma or laser W-NPs. After exposure, ITER-like W-NPs solutions were removed, and cells were lysed with total glutathione or oxidized glutathione lysis reagent. Luciferin detection regent was then added, and plates were further incubated for 15 min before luminescence was read at a GloMax^®^ Explorer Multimode Microplate Reader (Promega; Charbonnières-les-Bains, France). Data were analyzed by subtracting the GSSG reaction signal from the total glutathione to obtain the value of reduced glutathione in the sample. Data were expressed as % GSH/GSSG ratio ± SEM. Exposed cells were compared to the untreated ones.

### 2.7. Epigenotoxic Effects of W-NPs

We investigated if pristine plasma and laser ITER-like W-NPs induced epigenetic effects, such as variation in the DNA methylation, in BEAS-2B cells. Cells were seeded onto pre-coated T25 flasks and cultivated under standard conditions until they reached 80% confluency. The BEAS-2B cultures were then exposed for 24 h to 1 and 5 µg/mL pristine plasma and laser ITER-like W-NPs, and untreated controls were also included in the experimental set-up. The collection of the samples was performed at different time points (Table 1).

BEAS-2B cells were collected as follows: cell culture medium was removed and cells were washed in PBS before being trypsinized and centrifuged (1200 rpm, 5 min, RT). The pellets were dried by aspiration and then stored at −20 °C before DNA methylation was investigated.

We analyzed the methylation of the most frequent repetitive DNA sequences found in the human genome. Alu and LINE are, respectively, short or long interspersed repeats found throughout the genome, i.e., either in highly condensed heterochromatin and transcriptionally inactive loci but also in less condensed regions containing expressed genes. Given this broad distribution, epigenetic status of these two types of repeats is considered as a surrogate marker of the global profile for a given cell. We also analyzed DNA methylation of Satellite 2 and 3 (Sat 2 and Sat 3) repeats, corresponding mainly to heterochromatin associated with centromeric regions and sensitive to exogenous stress. DNA methylation was determined after sodium bisulfite modification of genomic DNA. This method is based on the oxidative desamination of cytosines. Unmethylated cytosines are converted to uraciles while methylation cytosines are not converted, allowing the analysis of individual CGs in any given sequence after PCR amplification and deep sequencing. DNA was extracted from the different types of samples using the Qiagen DNA prep kit following manufacturer’s instructions. The DNA methylation protocol, data acquisition, and analysis has already been described [24]. Data are presented as % DNA methylation at different time points.

### 2.8. Statistical Analysis

Cell viability, primary DNA damage, and oxidative stress were statistically analyzed by one-way ANOVA with Sidak post-hoc test. Cytostasis, cellular replication, and micronuclei frequency were analyzed by Chi-square test. Statistical analysis was performed using GraphPad Prism version 8.1.2 for Windows (GraphPad Software; San Diego, CA, USA).

## 3. Results

### 3.1. ITER-Like Plasma and Laser W-NPs Physico-Chemical Characterization

A detailed physico-chemical characterization of the ITER-like plasma and laser W-NPs, in their powder form, was already presented [15,16,19] and is here summarized in Table 2.

The laser W-NPs with a smaller average size were also more oxidized than plasma ones (82% of W^4+^ + W^6+^ compared to 10% of W^6+^ in the case of plasma W-NPs).

The ITER-like plasma and laser W-NPs suspensions were prepared from stock suspension at the initial concentration of 2.5 mg/mL in tris(hydroxymethyl)aminomethane (TRIS) medium. The particle size (Table 3) was determined using Dynamic Light Scattering (DLS). In the case of plasma-derived W-NPs, the particle size in TRIS did not significantly change after hydrogenation and tritiation. When dispersed in LHC-9, plasma-derived W-NPs at t = 0 h showed a slight increase in their mean size and PDI after hydrogenation and tritiation. In the case of laser ITER-like W-NPs dispersed in LHC-9, no significant differences were detected between the three types of laser W-NPs investigated, not in terms of size or in particle stability (PDI and Z pot). While dispersed in TRIS at t = 0, the average size of the pristine laser ITER-like W-NPs is much larger than the TEM size (Table 2). This can suggest an aggregation effect in TRIS medium. The hydrogenation and tritiation tends to decrease the average size of the laser-derived W-NPs. Such size decrease is difficult to interpret but it might be due to a partial oxidative dissolution that, already within 2 h, took place when W-NPs were suspended in LHC-9 culture medium. Indeed, even if it was impossible to quantify the amount of dissolution in the case of laser ITER-like W-NPs due to a low amount of raw material, in the case of pristine plasma ITER-like W-NPs, the dissolved W fraction in mass corresponded to 9%, 23%, 48%, and 58% for t = 0, 2, 4, and 24 h, respectively, for a 100 µg/mL suspension. When diluted at 10 µg/mL, dissolution further increased at 10%, 50%, 60%, and 66% for t = 0, 2, 4, and 24 h, respectively. A detailed W-NPs dissolution protocol (using ICP-MS after filtration at 0.02 µm) and data analysis is available elsewhere [19].

### 3.2. Cytotoxic Effects

**Figure 1:**
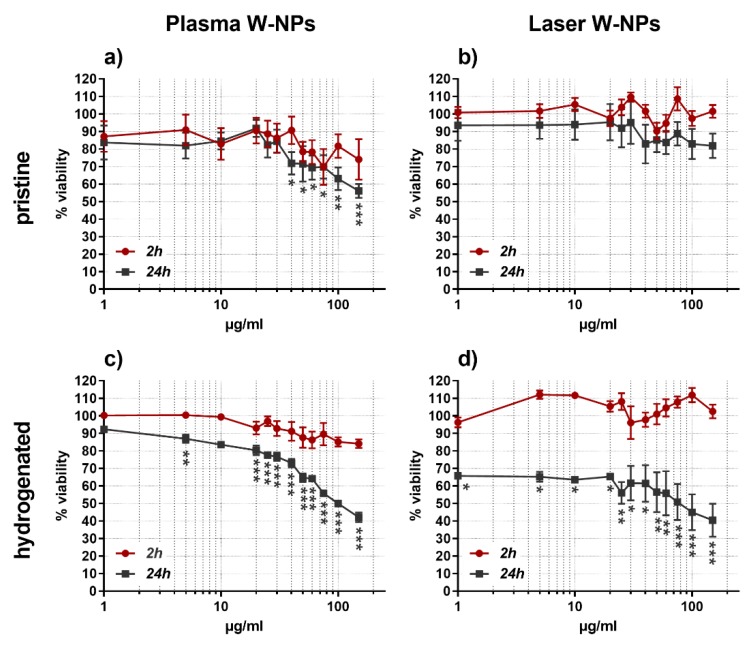
Cytotoxic effects exerted by W-NPs in BEAS-2B cells: (**a**) pristine plasma; (**b**) pristine laser; (**c**) hydrogenated plasma; (**d**) hydrogenated laser. At short exposures (2 h) none of the particles were able to affect the cell viability. After 24 h exposure, in contrast, only pristine laser W-NPs were not cytotoxic. Data are presented as mean % ± SEM of three independent experiments in triplicate. Statistical significance was evaluated by one-way ANOVA with Sidak post-hoc test: * *p* < 0.05; ** *p* < 0.01; *** *p* < 0.001.

At 2 h exposure none of the tested particles were able to significantly reduce the viability of BEAS-2B cells, whereas the behavior was different after 24 h incubation (Figure 1).

Plasma W-NPs, both pristine (Figure 1a) and hydrogenated (Figure 1c), severely affected the metabolic activity of BEAS-2B cells. The cytotoxic effect of pristine plasma W-NPs was statistically significant at concentrations ≥40 µg/mL and increased at the highest tested condition (150 µg/mL). Hydrogenated plasma W-NPs exhibited a statistically significant impairment of ATP production in BEAS-2B already at 5 µg/mL, which augmented in a concentration related manner.

While upon exposure to pristine laser W-NPs (Figure 1b) no cytotoxic effects were detected neither at 2 h nor at 24 h incubation time, the hydrogenated ones (Figure 1d) severely impaired the metabolism of BEAS-2B at 24 h exposure: 35% cytotoxicity was observed already at the lowest tested concentration of 1 µg/mL, and the effect was increased up to 60% cytotoxicity at 150 µg/mL.

Overall, our results show that at short exposures (2 h) W-NPs do not impair BEAS-2B cell viability, while longer incubations (24 h) have more significant effects. In addition, while at 24 h plasma W-NPs impaired the ATP production in BEAS-2B cells independently of their surface properties, the presence of hydrogen on laser W-NPs is necessary to induce cytotoxic effects. The more severe cytotoxicity observed upon exposure to hydrogenated particles could thus be due to a surface-effect. The presence of hydrogen enhances the cytotoxicity of ITER-like W-NPs and this effect seems time-related, since it does not appear at short exposures (2 h). Tritiated plasma and laser W-NPs could not be tested because of the lack of an appropriate plate reader in the facility where radioactive species are manipulated.

The cytotoxicity data allowed us to select some concentrations of interest for further experiments on the epigenotoxic effects of W-NPs. We thus chose 1–5–10–20 µg/mL as test concentrations because they do induce cytotoxicity without exceeding the levels (55% ± 5%) recommended for the genotoxicity tests [23].

### 3.3. Effects on Cytostasis and Cellular Replication

**Figure 2:**
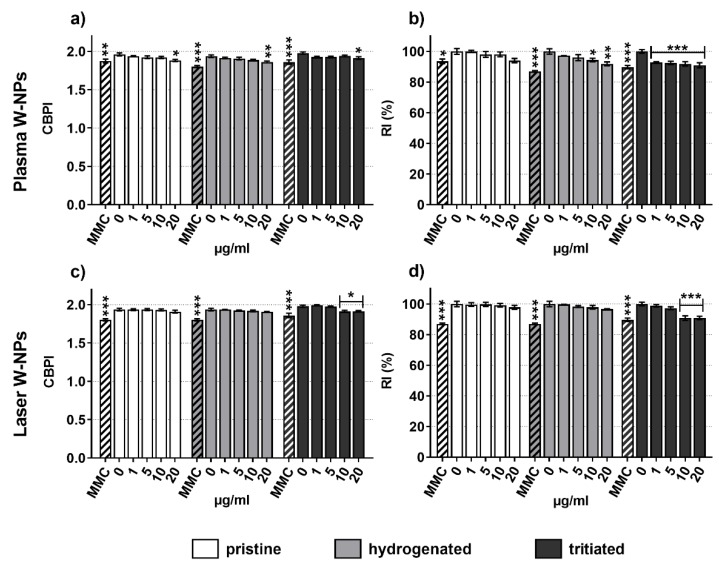
Cytostasis evaluation: (**a**) Cytokinesis Block Proliferation Index (CBPI) upon exposure to plasma W-NPs; (**b**) Replication Index (RI) upon exposure to plasma W-NPs; (**c**) CBPI following incubation with laser W-NPs; (**d**) RI following incubation with laser W-NPs. Independently of the presence or absence of hydrogen and tritium, both plasma and laser W-NPs reduced CBPI and RI of BEAS-2B cells. As expected, the positive control (Mitomycin C (MMC), 0.1 μg/mL) was both cytostatic and cytotoxic. Data are expressed as mean value ± SEM of three independent experiments, each in duplicate. Statistically significant differences from the untreated cells (0 µg/mL) were determined by Chi-square test: * *p* < 0.05, ** *p* < 0.01 and *** *p* < 0.001.

Figure 2 shows the cytostasis of BEAS-2B cells in the presence, for 24 h, of plasma (Figure 2a,b) and laser (Figure 2c,d) W-NPs in their pristine, hydrogenated, and tritiated forms. Cytostasis was evaluated via Cytokinesis-Block Proliferation Index (CBPI) and Replication Index (RI).

Both types of W-NPs impaired CBPI; nevertheless, no differences were observed comparing the results obtained upon plasma (Figure 2a) and laser (Figure 2c) exposure.

RI was also impaired by plasma and laser W-NPs. Pristine and hygdrogenated plasma (Figure 2b) and laser (Figure 2d) ITER-like W-NPs seem to behave similarly, while tritiated plasma W-NPs seem to exert damage to BEAS-2B cells at lower concentrations than their laser counterparts.

### 3.4. Micronuclei Formation

**Figure 3:**
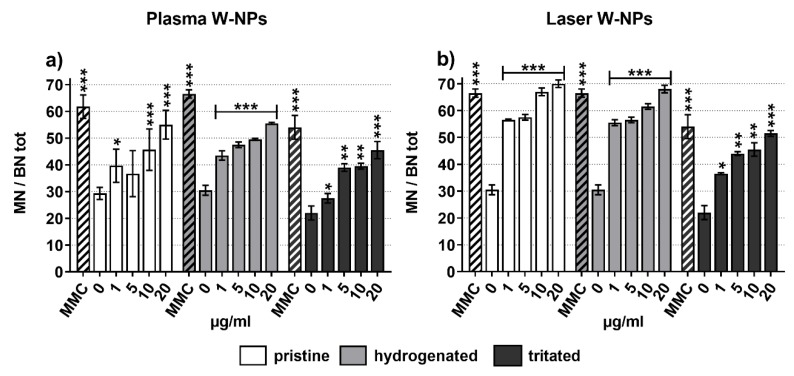
Micronuclei (MN) frequency in BEAS-2B cells exposed to (**a**) plasma W-NPs; (**b**) laser W-NPs. Both plasma and laser W-NPs, independently of the presence/absence of hydrogen and tritium, induced MN formation compared to the untreated cells. MMC (0.1 μg/mL) was used as positive control. Data are expressed as mean value ± SEM of two independent experiments, each in duplicate (*n* = 2000). Statistically significant differences from the untreated cells were determined by Chi-square test: * *p* < 0.05, ** *p* < 0.01 and *** *p* < 0.001.

The potential chromosomal damage in BEAS-2B cells was evaluated by CBMN assay (Figure 3). Dose-related and statistically significant increased MN frequency was measured following exposure to plasma (Figure 3a) and laser (Figure 3b) W-NPs in their pristine, hydrogenated, and tritiated forms. The presence of hydrogen and tritium did not enhance the genotoxic effect of plasma and laser W-NPs, as already observed for cytostasis and cellular replication (Figure 2).

To investigate more in detail the type of chromosomal damage exerted by the ITER-like plasma and laser W-NPs, a pancentromeric staining was performed (Figure S1). The aim was to distinguish between centromeric positive (CMpos) MN, issued by a whole chromosome loss exerted by an aneugenic compound, and centromeric negative (CMneg) MN, resulting from chromosome fragmentation following exposure to a clastogenic product.

As shown in Figure S1, plasma and laser W-NPs exert both CMpos and CMneg micronuclei. Compared to their respective negative controls, statistically significant CMpos and CMneg MN formation was observed. Since from the graphical representation it was not possible to clearly distinguish and understand which effect (clastogenic or aneugenic) was predominant in laser and plasma W-NPs, the CMpos and CMneg fold increase were calculated. In the presence of plasma W-NPs (Figure S1a,b) CMpos seem to be more induced than CMneg, suggesting a slight but not significant predominant aneugenic potential compared to the clastogenic one. Similarly, a slight predominance of CMpos MN can be observed in BEAS-2B cells upon exposure to laser (Figure S1c,d) W-NPs. Overall, the ITER-like plasma and laser W-NPs display both aneugenic and clastogenic potential.

### 3.5. DNA Damage Quantification

**Figure 4:**
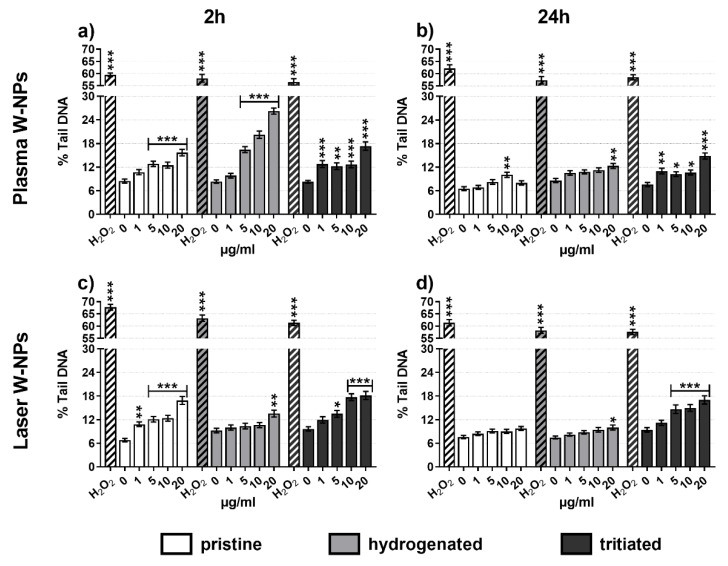
DNA damage by alkaline comet assay: (**a**) 2 h exposure to plasma W-NPs; (**b**) 24 h exposure to plasma W-NPs; (**c**) 2 h exposure to laser W-NPs; (**d**) 24 h exposure to laser W-NPs. Both plasma and laser W-NPs enhanced DNA strand breaks compared to the untreated cells (0 µg/mL) and displayed dose-related behavior. A total of 110 μM hydrogen peroxide (H_2_O_2_) was used as positive control. Data are presented as mean % tail DNA ± SEM of three independent experiments in triplicate. Statistical significance was evaluated by one-way ANOVA with Holm-Sidak post-hoc test: * *p* < 0.05; ** *p* < 0.01; *** *p* < 0.001.

To evaluate primary DNA lesions, the comet assay was performed following a conventional alkaline protocol. As shown in Figure 4, all the tested ITER-like W-NPs induced DNA fragmentation compared to the untreated BEAS-2B cells. While no differences in the quantification of the DNA damage were observed between tritiated plasma (Figure 4a,b) and laser (Figure 4c,d) at 2 h (Figure 4a,c) and 24 h (Figure 4b,d) exposure, the behavior of pristine and hydrogenated W-NPs was different. In fact, short incubation times generated more severe DNA damage than long exposures. This is very clear in cells exposed to pristine and hydrogenated plasma W-NPs and to pristine laser W-NPs, a bit less pronounced is the difference following incubation with hydrogenated laser W-NPs. The hypothesis to explain these results might be given by the formation of reactive oxidative species (ROS) that participate, as already reported in the literature, in DNA damage.

### 3.6. Oxidative Stress

**Figure 5:**
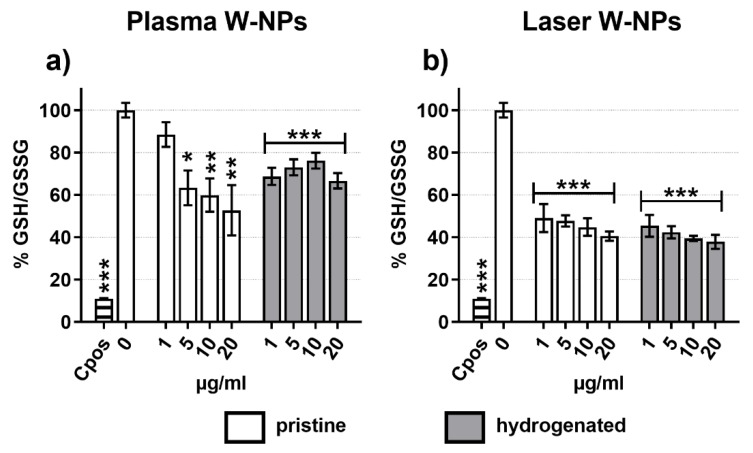
Oxidative stress induced by (**a**) plasma W-NPs; (**b**) laser W-NPs. Compared to the untreated cells (0 µg/mL) both plasma and laser W-NPs induced significant oxidative stress, as evaluated by the GSH/GSSG ratio. Menadione (20 µM) was used as positive control. Data are presented as mean % GSH/GSSG ± SEM of three independent experiments, each in triplicate (*n* = 9). Statistical significance was evaluated by one-way ANOVA with Holm-Sidak post-hoc test: * *p* < 0.05; ** *p* < 0.01; *** *p* < 0.001.

To verify our hypothesis that ROS play a role in the induction of DNA damage, we measured oxidative stress in BEAS-2B cells exposed to plasma (Figure 5a) and laser (Figure 5b) W-NPs. Already after 30 min exposure a highly statistically significant imbalance of the GSH/GSSG ratio was detected. With the exception of hydrogenated plasma W-NPs, which exerted the less severe reduction of GSH/GSSG ratio, pristine plasma W-NPs and the two tested types of laser W-NPs showed a dose-related oxidative stress. These data confirmed thus that oxidative stress took place upon exposure to ITER-like W-NPs and that it might trigger DNA damage.

Tritiated plasma and laser W-NPs could not be tested because of the lack of an appropriate plate reader in the radioactive facility. Nevertheless, because the hydrogenated particles can be considered as the non-radioactive equivalent of the tritiated ones, we could suppose that these latter ones have the same behavior than the hydrogenated.

### 3.7. DNA Methylation Changes Analysis

**Figure 6:**
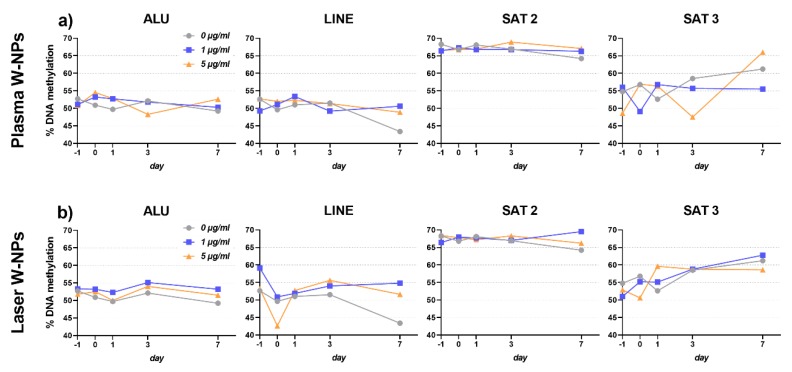
DNA methylation level determined after exposure of BEAS-2B cells to naked induced by pristine (**a**) plasma W-NPs; (**b**) laser W-NPs. No significant differences were observed at any of the time points of any of the BEAS-2B generations investigated. No differences could be observed between the effects exerted by plasma and laser ITER-like W-NPs.

Epigenetic variations were evaluated at different time points and in different BEAS-2B generations (F0 and F1) upon 24 h exposure to 1–5 µg/mL pristine plasma (Figure 6a) and laser (Figure 6b) ITER-like W-NPs. Compared to the untreated control, for the different sequences analyzed, no significant changes in DNA methylation were observed at any time point tested, with none of the sequences we used in our analysis. Additionally, no differences were observed in the % of methylated DNA in cells exposed to plasma or laser ITER-like W-NPs, confirming again that the biological effects of these two types of particles are similar despite their physico-chemical differences.

## 4. Discussion

When thermonuclear fusion reactors become operational, generating tritiated tungsten particles, they could represent a potential risk for the environment and human health as they might be released in case of LOVA. Since the reactor is still under construction, we can only gather preliminary information from the current literature, which is mainly related to different types and forms of W and not to ITER-derived W-NPs.

The aim of our study was thus to provide an evaluation of the cytotoxic, genotoxic, and epigenetic in vitro effects that ITER-like W-NPs might have on human health, in relation with the possible W-NPs transformation in biological media. In particular, we focused our attention to an in vitro 2D model of the lung, the BEAS-2B cell line, that was chosen because inhalation is the main route of exposure to W. Similarly, a significant number of toxicity studies on W were performed on lung-derived cell lines [4,5,7,8,10,12].

The main outcome of the current literature is that W is cytotoxic and induces apoptosis. In addition, W can exert MN formation or DNA damage as well as alter gene expression and arrest the cell cycle. Generally, the production of ROS is considered as the main cause triggering W genotoxicity. Analogously, our data showed that plasma and laser ITER-like W-NPs exert cytotoxic and genotoxic effects in BEAS-2B cells, and the increased oxidative stress might be one of the reasons explaining our results.

Even if we can identify similarities between the outcome of previous studies on W and our data, there are some important differences that we think are pivotal in understanding our results and the mechanisms by which W results toxic. First of all, our bench produced plasma and laser ITER-like W-NPs display peculiar physico-chemical properties [15,16,17]. More in detail, although they are metallic W-NPs, XAS and helium pycnometer analysis have shown that a significant fraction of tungsten oxide (WO_x_) is present and it could participate in the toxicity of the particles. WO_3_ represented only a small fraction (10%) of plasma ITER-like W-NPs, while more oxidation was observed on laser ITER-like W-NPs powders (78% WO_3_ by helium pycnometer and 50% WO_3_ + 32% WO_2_ by XAS analysis). Additionally, ITER-like W-NPs are highly soluble. ICP-MS, in fact, revealed that the dissolved W fraction in 100 µg/mL suspensions corresponded, in LHC-9 culture medium, to 9% at t = 0 h and it increased up to 23% and 58% at, respectively, 2 and 24 h incubation [17]. As expected, further dilution of W suspension to 10 µg/mL enhanced their dissolution kinetic: 10% was the soluble W fraction measured at t = 0 h, while 50% and 66% was dissolved at t = 2–24 h [17]. All together these data indicate that the ITER-like W-NPs we investigated were characterized by oxidation and dissolution. These findings bring thus a new perspective on the toxicity of ITER-like W-NPs and they make the plasma and laser particles we investigated unusual.

Not much information is reported in the literature on the solubility of W-containing particles, on their oxidation, and on the involvement of ionic W and W oxides in exerting toxicity. In contrast, studies on the soluble Na_2_WO_4_ and the on WO_3_NP have already been reported. Sodium tungstate was applied to BEAS-2B cells at concentrations ranging from 50 to 250 µM in order to investigate the carcinogenicity of W via several endpoints such as cell transformation, cell migration, and the activation of multiple cancer-related pathways [4]. After 6 weeks of treatment, results clearly showed that Na_2_WO_4_ increased BEAS-2B colonies formation at all tested doses; in addition, cell migration was tested via the cell scratch assay and the transformed clones were able to heal the wound within less than a day after the scratch was made. Changes in gene expression due to tungstate exposure were observed in BEAS-2B transformed clones: RNA sequencing showed that more than 16,000 genes were altered and the majority of them were related to lung cancer and leukemia, inflammation, and tumor morphology. Overall, the results of Laulicht and coauthors showed that ionic W has carcinogenic in vitro potential [4].

In freshly isolated human peripheral blood lymphocytes (hPBL) Na_2_WO_4_ was applied in the range 0.1–1–10 mM for 24–96 h and apoptosis, cell cycle, and cytokines secretions were investigated [3]. Early apoptosis increased in tungstate exposed hPBL whereas the number of lymphocytes entering the cell cycle was reduced. Already at relatively low concentration (1 mM), W in its ionic form significantly increased the G_0_/G_1_ fraction and impaired the number of cells in S- or G_2_/M-phase. Moreover, when 5-ethynyl-2′-deoxyuridine (EdU) was used to test DNA synthesis, tungstate was observed to reduce, up to 50%, the EdU-positive cells, also those in G_0_/G_1_-phase. Likewise, the production of cytokines such as TNF-α, IL-10, and IL-6 resulted significantly lowered, probably due to the high rate of apoptotic hPBL upon exposure to Na_2_WO_4_. Osterburg and coauthors suggested that the mechanism by which ionic W induces toxic effects depends on its ability to inhibit phosphatase and alter the phosphorylation of some intracellular molecules [3].

From the literature, ionic W seems thus to induce a variety of effects on different in vitro cellular systems. This effect is confirmed by our results on the soluble plasma and laser ITER-like W-NPs that, at least after 24 h exposure, decreased viability and, even if not in a significant manner, the cytostasis of BEAS-2B cells. If we only consider the ionic W released by plasma and laser W-NPs as the key factor triggering cytotoxicity, we should keep in mind that the differences in cell viability observed at 2 and 24 h exposure might depend on the kinetic of dissolution of the particles. In fact, the amount of ionic W measured by ICP-MS was low at 2 h (9%–10%), but much higher (58%–66%) at 24 h. We thus suggest this is one of the reasons that allowed us to observe more cytotoxic effects at long than at short exposures (Figure 1).

Since the presence of WO_2_ and WO_3_ we detected during powders characterization is not negligible [24], oxidation should also be considered when analyzing plasma and laser ITER-like W-NPs toxicity. Ex vivo bone cells and hepatocytes of 8 weeks old male Sprague-Dawley rats were used to investigate the cytotoxicity, the genotoxicity, and the mutagenic potential of WO_3_NPs [25,26]. Upon 30 days of intraperitoneal injections of WO_3_NPs at the dose of 25–50–100 mg/kg b.w., bone cells were collected from tibia. While chromosome aberration test did not show any difference in bone cells from exposed rats compared to the untreated controls, mitotic index, a marker for cytotoxicity, and MN frequency, an indicator of genotoxic damage, were affected by WO_3_NPs. Mitotic index was significantly decreased at all the tested doses; MN frequency, conversely, was enhanced only in rats exposed to 50–100 mg/kg b.w. [25]. Turkez and coauthors further investigated the effects of WO_3_NPs in rat hepatocytes [26]. After performing an ex vivo culture of hepatocytes, cells were exposed for 72 h to WO_3_NPs concentrations ranging from 5 to 1000 ppm. The cytotoxic potential was detected by observing a decrease in cell viability, assayed by 3-(4,5-dimethylthiazol-2-yl)-2,5-diphenyltetrazolium bromide (MTT) test, and an increase in the levels of extracellular lactate dehydrogenase at fairly high concentrations (≥300 ppm). Surprisingly, no MN formation was observed at any of the tested WO_3_NPs concentrations, while oxidative stress and secondary oxidative DNA damage were reported [26]. From the publications of Turkez and coauthors [25,26] it seems that WO_3_NPs are feebly cytotoxic and could induce chromosome losses. They also generate significant oxidative stress that can cause indirect DNA damage via the formation of adducts, as observed via 8-oxo-2-deoxyguanosine quantification or impair mitotic spindle.

In contrast, plasma and laser ITER-like W-NPs showed significant cytotoxicity on BEAS-2B cultures, they resulted genotoxic by significantly enhancing the MN frequency and by inducing DNA strand breaks. In agreement with previous studies [25,26], we equally observed oxidative stress that might have triggered DNA damage. Nevertheless, the differences between our results and those of Turkez and coauthors [25,26] might be due to the fact that only a fraction of our ITER-like W-NPs was oxidized. We think that both oxidation and dissolution played a synergistic role in the initiation of ITER-like W-NPs toxicity. Since the current literature on WO_3_NPs does not provide information on the particle dissolution, further comparison cannot be done between the studies of Turkez et al. [25,26] and ours.

While we do not have any physico-chemical information on the WO_3_NPs used in the Sprague-Dawley ex vivo studies presented just above [25,26], Chinde and colleagues have presented a thorough characterization of the WO_3_NPs they have tested on the alveolar A549 cell line [5]. In milliQ water, by TEM, the mean size of WO_3_NPs corresponded to 54 ± 29 nm, while DLS corresponded, respectively, to 170 ± 2 nm and 224 ± 5 nm in milliQ and in DMEM cell culture medium. WO_3_NPs are thus slightly bigger than the plasma and laser ITER W-NPs we investigated, whose size range, via DLS and in LHC-9 cell culture medium, was set to 103–135 nm and to 155–174 nm, respectively.

Chinde and coauthors have also investigated the dissolution of WO_3_NPs in cell culture medium. Particles were suspended for 24–48 h in DMEM and the nominal WO_3_ concentration was compared to total W measured by ICP-MS. While the nominal WO_3_ concentration of 200 and 300 µg/mL corresponded to 170 and 330 µg/mL total W concentration before ultracentrifugation, only a small fraction of soluble W (0.9 and 2.4 µg/mL) was detected in ultracentrifuged solutions [5]. These results clearly indicate that Chinde et al. used non-soluble WO_3_NPs; conversely, our ITER-like W-NPs showed a significant dissolved W mass fraction already at t = 0 h in cell culture medium which further increased up to 24 h (Table 2).

Our results on BEAS-2B cells confirm the in vitro lung toxicity of WO_3_NPs observed by Chinde and coauthors, although some differences in the experimental set-up and in its outcome are noticeable. In A549 cells, the cytotoxicity was observed at high concentrations (≥200 µg/mL WO_3_NPs; 24–48 h exposure duration) [5], whereas in BEAS-2B cells and at 24 h exposure, plasma and laser ITER-like W-NPs resulted cytotoxic already at 1 µg/mL. Similarly, DNA damage, MN frequency, apoptosis, and oxidative stress were only enhanced in A549 cells exposed at nominal concentrations ≥200 µg/mL WO_3_NPs [5]. Moreover, WO_3_NPs uptake quantification showed that only 0.3% of particles were internalized by A549 cells, and that the most significant fraction (70%–75%) of W was diluted in the supernatant.

The nominal concentrations tested and the oxidation state of the particles represent the greater differences between the study of Chinde et al. and ours. Nominal WO_3_NPs concentrations of at least 200 µg/mL, in fact, differ by 10-fold compared to the maximal plasma and laser ITER-like W-NPs concentration we tested (20 µg/mL). Additionally, plasma and laser ITER-like W-NPs contained, respectively, 10% WO_3_ and 32% WO_2_/50% WO_3_, which further decreases the nominal concentration of WO_x_ we applied to BEAS-2B cells. Since only a fraction of the plasma and laser ITER-like W-NPs powders is oxidized on their surface, our hypothesis is that the oxidation and the oxidation state are not the unique factors inducing cytotoxic and genotoxic effects in BEAS-2B cells.

To our knowledge, very few epigenetic investigations have been performed on various forms of W. Verma and co-workers investigated by Western blot the posttranslational histone epigenetic modifications, such as acetylation of histone 3 (H3), phosphorylation of Ser10 (H3-Ser10), and the trimethylation of histone H3 on lysine 4 (H3K4me3) in the tail of histone H3 in human embryonic kidney (HEK293), human neuroepithelioma (SKNMC), mouse myoblast (C2C12), and hippocampal primary neuronal cultures exposed to 50–184 µg/mL metal W and W-alloys (91% W, 6% Ni, 3% Co) for 1 day or 1 week, depending on the cell type [27]. H3K4me3 showed no changes in any of the cell cultures following metal exposure, indicating absence of modification in transcriptional activation of genes. Whereas W induced dephosphorylation of H3-Ser10 exclusively in HEK293 cells, tungsten-alloy reduced H3-Ser10 phosphorylation in C2C12 cells and hippocampal primary neuronal cultures, probably taking advantage of the synergistic toxic effects of W with Ni and Co [27]. H3K4me3, as well as the demethylation of histone H3 at lysine residue 9 (H3K9me2), were further investigated in BEAS-2B and A549 cells exposed, in vitro, to 1–2.5–5–10 mM Na_2_WO_4_ for 48 h [28]. Via Western blot analysis Laulicht-Glick and coauthors observed that global levels of both H3K4me3 and H3K9me2 increased compared to the untreated controls, and that the methylation was not reversible when the treatment solutions were removed and cells further incubated with cell culture medium. Further investigation revealed that the degradation of the demethylases JMJD1A and JARID1A caused the increased H3 methylation in BEAS-2B and A549 [28].

Conversely to Verma et al. [27] and to Laulicht-Glick et al. [28], we quantified the variations in DNA methylation of BEAS-2B cells upon 24 h exposure to 1–5 µg/mL plasma and laser ITER-like W-NPs. Under our experimental conditions we did not detect changes in DNA methylation compared to the control cells, not at any of the days post-exposure nor in sub-cultured BEAS-2B cells (F1 generation). Additional tests might be required, extending the exposure period or increasing the test concentrations, to verify if and at which time point epigenetic effects are induced by ITER-like W-NPs.

## 5. Conclusions

Despite its use, W still represents a potential occupational or accidental risk. Even if, upon intake, the human body rapidly excretes W, traces could be found in kidney, liver, bones, and spleen [29]. The Occupational Safety and Health Administration (OSHA) has established that 5 and 1 mg/m^3^ are the permissible exposure limits for, respectively, insoluble and soluble W compounds employed in the construction and shipyard industries [30,31]. Nevertheless, no information is yet available on ITER-derived W-NPs, their morphology, their physico-chemical properties, and their biological profile. All together, these facts highlight the importance of our study attempting at fulfilling the gap of knowledge on ITER W-NPs. Under our experimental conditions ITER-like plasma and laser W-NPs induced, in a 2D in vitro model of the respiratory compartment, cytotoxic and genotoxic effects as well as strong oxidative potential. Our data represent thus a first, although not exhaustive, multi-endpoint characterization of the biological profile and of the potential risk that ITER-released W-NPs might induce on human health.

## Figures and Tables

**Table 1:** Experimental set-up for epigenetic sampling preparation.

Day −1	Cells collected before exposure (generation F0)
Day 0	Cells collected at the time of their exposure to plasma and laser ITER-like W-NPs (generation F0)
Day 1	Cells collected at the end of the 24 h exposure (generation F0)
Day 3	Cells collected 2 days after the end of the exposure (generation F0)
Day 7	Day 3 cells passaged and collected at day 6 after the end of the exposure (generation F1)

**Table 2:** Summary of the physico-chemical properties of plasma and laser tungsten nanoparticles (W-NPs) powders.

**Plasma W-NPs**		
*Synthesis*	Magnetron Sputtering	
*Electron microscopy*	Scanning electron microscopy	100–200 nm mean size
	Transmission electron microscopy	Inhomogeneous shape: star-like, squared and round
*Crystalline structure*	Fourier transform pattern	Beta-phase W metal
	XRD	Beta-phase W metal
*Density*	Helium pycnometer	14.32 g/cm^3^
*Surface area*	BET	4 m^2^/g
*W/WO_x_ ratio (in mass)*	Helium pycnometer	90% W metal, 10% WO_3_
	XAS	90% W metal, 10% WO_3_
**Laser W-NPs**		
*Synthesis*	High energy laser ablation	
*Electron microscopy*	Transmission electron microscopy	60–80 nm mean size
*Crystalline structure*	XRD	Alpha-phase W metal
*Density*	Helium pycnometer	8.27 g/cm^3^
*Surface area*	BET	43.5 m^2^/g
*W/WO_x_ ratio (in mass)*	Helium pycnometer	22% W metal, 78% WO_3_
	XAS	18% W metal, 32% WO_2_, 50% WO_3_

**Table 3:** Size and zeta potential determination of International Thermonuclear Experimental fusion Reactor (ITER)-like plasma and laser W-NPs in Tris-HCl and in complete BEAS-2B culture medium (LHC-9 medium).

t = 0 h 100 µg/mL	Plasma-Derived W-NPs			Laser-Derived W-NPs		
	Mean Size (nm)	PDI *	Z Pot (mV)	Mean Size (nm)	PDI	Z Pot (mV)
***TRIS buffer***						
***Pristine***	122	0.016	−44	314	0.290	−24
***Hydrogenated***	107	0.090	−39	265	0.330	−32
***Tritiated***	126	0.120	−31	284	0.301	−27
***LHC-9 culture medium***						
***Pristine***	103	0.116	−8	162	0.400	−7
***Hydrogenated***	122	0.250	−10	155	0.350	−11
***Tritiated***	135	0.210	−13	174	0.500	−10

* PDI: Polydispersity Index.

## Supplementary Materials

The following are available online at https://www.mdpi.com/2079-4991/9/9/1233/s1, Figure S1: Pancentromeric staining in BEAS-2B cells exposed to W-NPs: (**a**) CMpos and CMneg formation upon exposure to plasma W-NPs; (**b**) CMpos and CMneg fold increase compared to untreated cells upon exposure to plasma W-NPs; (**c**) CMpos and CMneg formation upLHCon exposure to laser W-NPs; (**d**) CMpos and CMneg fold increase compared to untreated cells upon exposure to laser W-NPs. Independently of the presence/absence of hydrogen and tritium, ITER-like plasma, and laser W-NPs induced both CMpos and CMneg MN formation compared to the untreated cells (0 µg/mL). MMC (0.1 μg/mL) was used as positive control. Data are expressed as mean value ± SEM of two independent experiments, each in duplicate. Statistically significant differences from the untreated cells were determined by Chi-square test: * *p* < 0.05, ** *p* < 0.01 and *** *p* < 0.001.
