# Supplementary material for: In Vitro Analysis of the Effects of ITER-Like Tungsten Nanoparticles: Cytotoxicity and Epigenotoxicity in BEAS-2B Cells
Source: Nanomaterials (Basel). 2019 Aug 30;9(9):1233. doi: 10.3390/nano9091233 (PMC6780084; doi:10.3390/nano9091233)
Supplement: Supplementary file 1 [file nanomaterials-09-01233-s001.pdf]

SupplementaryMaterials

In Vitro Analysis of the Effects of ITER-Like Tungsten Nanoparticles: Cytotoxicity and Epigenotoxicity in BEAS-2B Cells

Chiara Uboldi [1], Marcos Sanles Sobrido [2], Elodie Bernard [3,4], Virginie Tassistro [1], Nathalie Herlin-Boime [5], Dominique Vrel [6], Sébastien Garcia-Argote [7], Stéphane Roche [8], Fréderique Magdinier [8], Gheorghe Dinescu [9], Véronique Malard [4], Laurence Lebaron-Jacobs [4], Jerome Rose [2], Bernard Rousseau [7], Philippe Delaporte [3], Christian Grisolia [10] and Thierry Orsière [1,*]

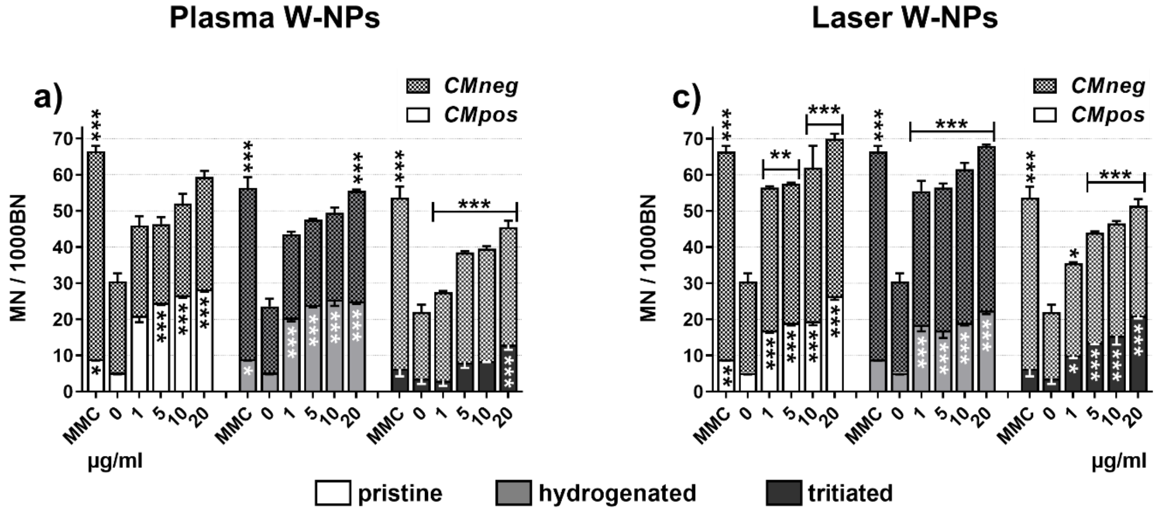

b)

Fold increase	CMpos	CMneg	CMpos	CMneg	CMpos	CMneg
	Pristine		*Hydrogenated*		*Tritiated*	
**MMC**	1.71	2.28	1.71	2.58	1.72	2.58
**1 µg/ml**	4.00	1.00	3.90	1.25	1.00	1.34
**5 µg/ml**	4.69	1.00	4.57	1.28	2.18	1.66
**10 µg/ml**	5.08	1.00	4.86	1.31	2.32	1.69
**20 µg/ml**	5.39	1.23	4.76	1.66	3.54	1.77

d)

Fold increase	CMpos	CMneg	CMpos	CMneg	CMpos	CMneg
	Pristine		*Hydrogenated*		*Tritiated*	
**MMC**	1.71	2.28	1.71	2.28	1.72	2.58
**1 µg/ml**	3.24	1.56	3.52	1.46	2.72	1.39
**5 µg/ml**	3.62	1.52	3.24	1.56	3.68	1.66
**10 µg/ml**	3.71	1.68	3.62	1.68	4.22	1.69
**20 µg/ml**	5.05	1.72	4.28	1.80	5.72	1.66

Figure S1. Pancentromeric staining in BEAS-2B cells exposed to W-NPs: (**a**) CMpos and CMneg formation upon exposure to plasma W-NPs; (**b**) CMpos and CMneg fold increase compared to untreated cells upon exposure to plasma W-NPs; (**c**) CMpos and CMneg formation upon exposure to laser W-NPs; (**d**) CMpos and CMneg fold increase compared to untreated cells upon exposure to laser W-NPs. Independently of the presence/absence of hydrogen and tritium, ITER-like plasma and laser W-NPs induced both CMpos and CMneg MN formation compared to the untreated cells (0 µg/ml). MMC (0.1 µg/mL) was used as positive control. Data are expressed as mean value ± SEM of two independent experiments, each in duplicate. Statistically significant differences from the untreated cells were determined by Chi-square test: *$p < 0.05$, **$p < 0.01$ and ***$p < 0.001$.
